# Supplementary material for: Comparative multiomics analysis of cell physiological state after culture in a basket bioreactor
Source: Sci Rep. 2022 Nov 23;12:20161. doi: 10.1038/s41598-022-24687-4 (PMC9686226; doi:10.1038/s41598-022-24687-4)

3666056.1:39637725:39637805:+@NW_023666056.1:39688221:39688272:+@NW_023666056.1:39702161:3970[illegible]

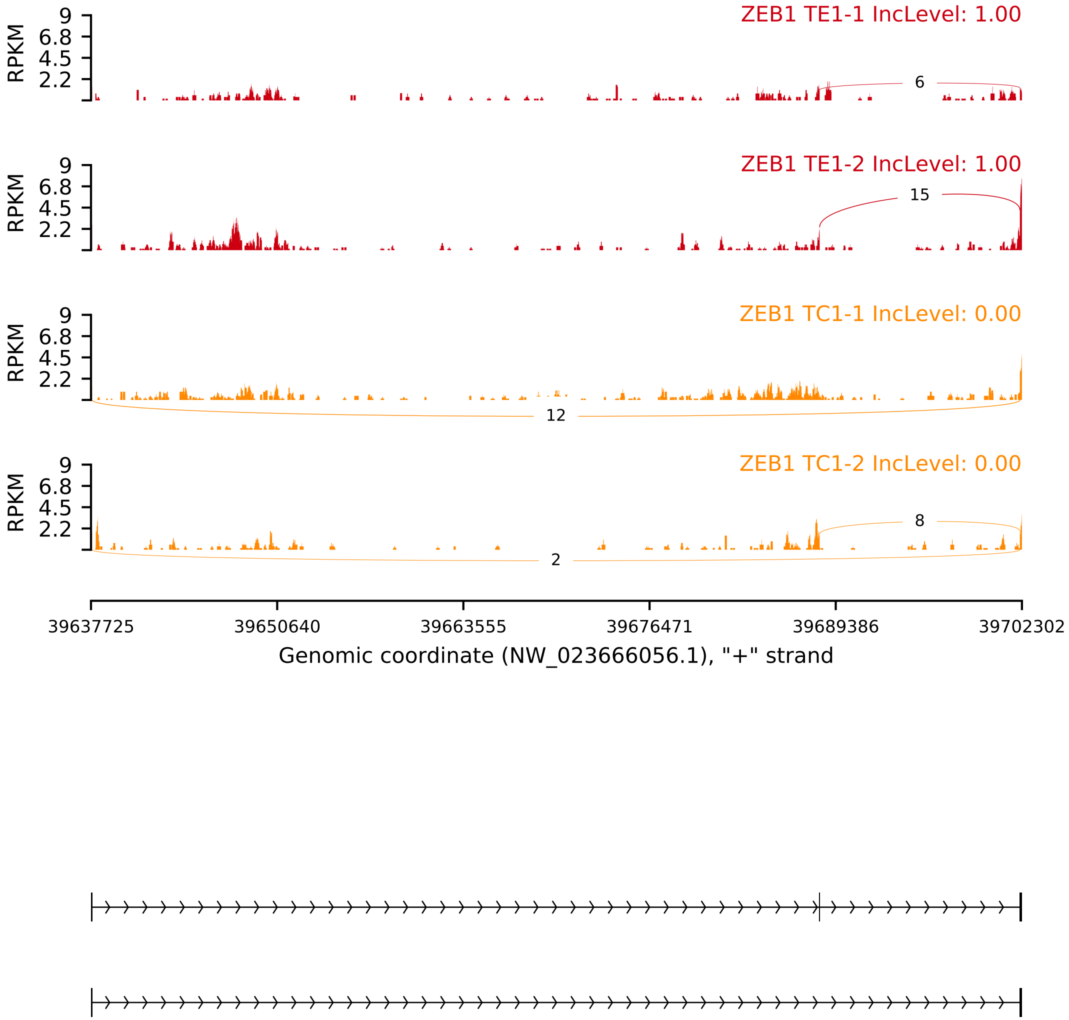

Supplement: Supplementary file 1 — Supplementary Information 1. [file 41598_2022_24687_MOESM1_ESM.zip › raw data/Transcriptomic raw data/7.AS/2.dasplot/TE1vsTC1/SE/16_ZEB1_NW_023666056.1_39637725_39637805_+@NW_023666056.1_39688221_39688272_+@NW_023666056.1_39702161_39702303_+.pdf]
